# Changes in Tachycardia Cycle Length with Varying Degrees of Right Bundle Branch Block: What Is the Mechanism?

**DOI:** 10.19102/icrm.2022.130801

**Published:** 2022-08-15

**Authors:** Jefferson Jaber, Alessandro Amaral, Guilherme Fenelon

**Affiliations:** ^1^Department of Cardiology, Hospital Santa Marcelina, São Paulo, Brazil; ^2^Arrhythmia Center, Hospital Israelita Albert Einstein, São Paulo, Brazil

**Keywords:** Aberrant conduction, right bundle branch block, supraventricular tachycardia

## Abstract

The present case contributes to understanding the mechanism and differential diagnosis of a wide QRS complex tachycardia with varying degrees of right bundle branch block morphology.

## Case presentation

**Figure 1:**
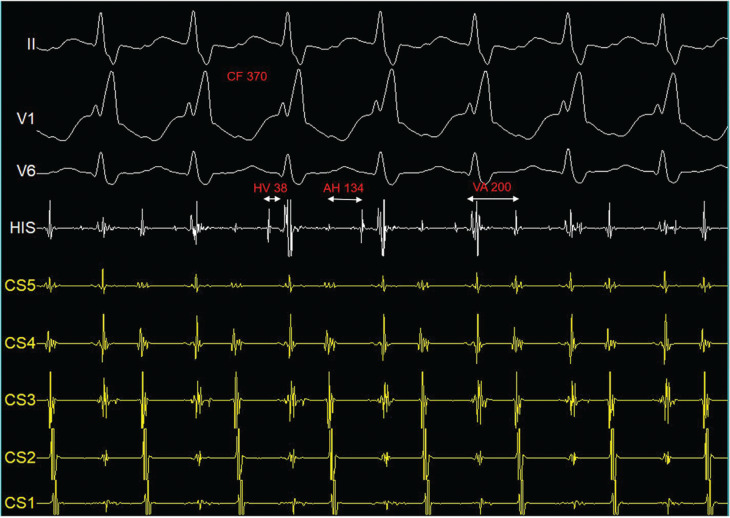
Tachycardia with a right bundle branch block morphology. From top to bottom: electrocardiogram leads II, V1, and V6; intracardiac recordings His bundle (His [distal]); and coronary sinus from distal (CS5) to proximal (CS1). Paper speed, 100 mm/s. *Abbreviation:* CF, cycle length.

**Figure 2:**
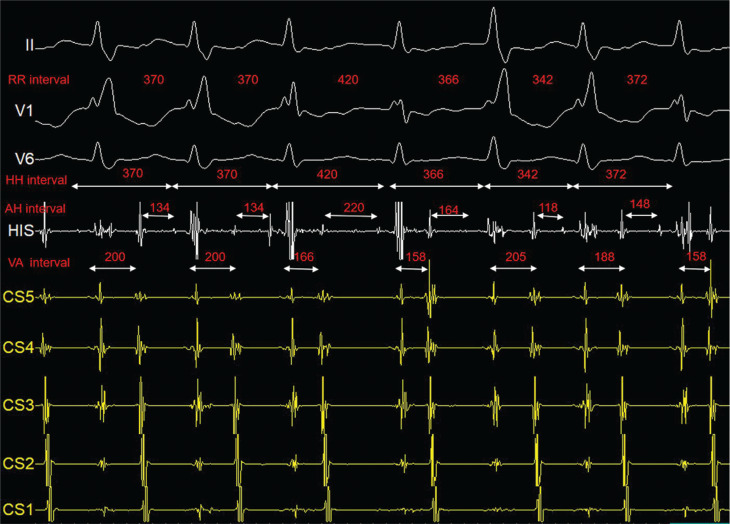
Tachycardia with varying degrees of right bundle branch block morphology associated with changes in tachycardia cycle length. From top to bottom: electrocardiogram leads II, V1, and V6; intracardiac recordings His bundle (His [distal]); and coronary sinus from distal (CS5) to proximal (CS1). Paper speed, 100 mm/s. *Abbreviation:* CF, cycle length.

A healthy 24-year-old man with recurrent palpitations was referred for an electrophysiology study and possible radiofrequency catheter ablation. A resting electrocardiogram taken during sinus rhythm was normal. During catheter positioning, a regular wide QRS complex tachycardia (cycle length, 370 ms) with a right bundle branch block (RBBB) morphology and a septal ventriculoatrial (VA) interval of 200 ms was induced **(Figure 1)**. During tachycardia, the QRS complexes showed varying degrees of RBBB morphology associated with changes in the tachycardia cycle length **(Figure 2)**. Ventricular overdrive pacing maneuvers interrupted the tachycardia. What is the mechanism of the tachycardia, and what is the explanation for the changes in tachycardia cycle length?

## Discussion

The differential diagnosis of a regular wide QRS complex tachycardia with a typical RBBB morphology and 1:1 VA conduction includes ventricular tachycardia arising from the left ventricle, pre-excited tachycardia mediated through a left-sided accessory pathway, and supraventricular tachycardia with RBBB (pre-existing or rate-related) aberrancy. During tachycardia, the His-bundle electrogram precedes each QRS complex with an H–V interval of 38 ms, the same as in sinus rhythm. These features indicate ventricular activation through the His–Purkinje system, thus excluding pre-excited tachycardia and ventricular tachycardia as possible mechanisms. As QRS complexes were normal during sinus rhythm, the diagnosis of a supraventricular tachycardia with rate-related RBBB aberrant conduction was made.^1^

A supraventricular tachycardia with RBBB aberrancy can be caused by atrioventricular nodal re-entrant tachycardia (AVNRT), atrial tachycardia (AT), and orthodromic atrioventricular re-entry tachycardia (AVRT) mediated by a concealed accessory pathway. During tachycardia, the septal V–A interval was 180 ms, excluding typical (slow–fast) AVNRT, which is characterized by short V–A intervals (<70 ms). In addition, the sequence of retrograde atrial activation in the coronary sinus was proximal to distal, excluding a concealed left-sided accessory pathway.^1^

Of note, the spontaneous variations in the degree of RBBB morphology preceding changes in the VA interval and tachycardia cycle length exclude all types of AVNRT (slow–fast, fast–slow, slow–slow) and AT. The development of bundle branch block during AVNRT should not modify the tachycardia cycle length because the re-entrant circuit is confined to the atrioventricular (AV) node and perinodal atrial tissue. Finally, AT can be ruled out by the observation that V–A interval variations precede and predict changes in atrial cycle length.^2,3^

An increase in the VA interval and tachycardia cycle length with the development of bundle branch block is a diagnostic finding for orthodromic AVRT mediated by an ipsilateral accessory pathway. Orthodromic AVRT is a macro–re-entrant circuit in which the antegrade limb is formed by the AV node and His–Purkinje system and the retrograde limb by the accessory pathway. The occurrence of bundle branch block on the same side of the pathway results in a slow-down of the tachycardia because the wavefront must travel through the contralateral bundle and then transseptally to the ipsilateral bundle, thus lengthening the reentrant circuit size.^2,3^

In the present case, changes in the V–A interval and tachycardia cycle length were preceded by varying degrees of RBBB. This phenomenon started in the third QRS complex **(Figure 2)**, where partial recovery of the right bundle branch conduction shortened the V–A interval (from 200 to 166 ms), resulting in an abrupt prolongation of the following A–H interval (from 134 to 220 ms). This prolongation of the A–H interval is associated with an increase in the H–H interval (from 370 to 420 ms), allowing complete recovery of right bundle branch conduction and further shortening of the V–A interval (from 166 to 158 ms). Partial recovery of AV node excitability occurs, and the A–H interval shortens from 220 to 164 ms. The subsequent long–short sequence in the H–H interval (from 420 to 366 ms) promotes complete right bundle branch aberrancy (note the prolonged QRS duration), resulting in a maximal increase of the V–A interval (205 ms). In the next cycles, there is variation in the A–H and V–A intervals associated with progressive recovery of right bundle conduction (note progressive shortening of QRS duration). Therefore, changes in the tachycardia cycle length were produced by the V–A interval variation promoted by varying degrees of RBBB morphology, which in turn caused variation in the A–H interval.^2,3^ Despite the sudden changes in the A–H interval during tachycardia, dual A–H nodal physiology was not demonstrated. This finding may be related to AV nodal accommodation.

This patient had an orthodromic reciprocating tachycardia mediated by a right lateral accessory pathway. Radiofrequency ablation at this location was successfully performed.
